# Differences in tumor microenvironments between primary lung tumors and brain metastases in lung cancer patients: therapeutic implications for immune checkpoint inhibitors

**DOI:** 10.1186/s12885-018-5214-8

**Published:** 2019-01-07

**Authors:** Ryul Kim, Bhumsuk Keam, Sehui Kim, Miso Kim, Se Hyun Kim, Jin Wook Kim, Yu Jung Kim, Tae Min Kim, Yoon Kyung Jeon, Dong-Wan Kim, Doo Hyun Chung, Jong Seok Lee, Dae Seog Heo

**Affiliations:** 1 Department of Internal Medicine, Seoul National University Hospital, 101 Daehak-ro, Jongno-gu, Seoul, 03080 Republic of Korea; 2 Cancer Research Institute, Seoul National University College of Medicine, 101 Daehak-ro, Jongno-gu, Seoul, 03080 South Korea; 3 Department of Pathology, Seoul National University Hospital, 101 Daehak-ro, Jongno-gu, Seoul, 03080 South Korea; 4 Department of Internal Medicine, Seoul National University Bundang Hospital, 82 Gumi-ro 173 Beon-gil, Bundang-gu, Seongnam-si, Gyeonggi-do 13620 South Korea; 5 Department of Neurosurgery, Seoul National University Hospital, 101 Daehak-ro, Jongno-gu, Seoul, 03080 South Korea

**Keywords:** Immunotherapy, Lung cancer, Brain metastasis, PD-1, PD-L1

## Abstract

**Background:**

We aimed to compare intra- and extracranial responses to immune checkpoint inhibitors (ICIs) in lung cancer with brain metastases (BM), and to explore tumor microenvironments of the brain and lungs focusing on the programmed cell death-1 (PD-1)/programmed cell death ligand-1 (PD-L1) pathway.

**Methods:**

Two cohorts of lung cancer patients with BM were analyzed. Cohort 1 included 18 patients treated with nivolumab or pembrolizumab, and intra- and extracranial responses were assessed. Cohort 2 comprised 20 patients who underwent both primary lung surgery and brain metastasectomy. Specimens from cohort 2 were subjected to immunohistochemical analysis for the following markers: CD3, CD4, CD8, FOXP3, and PD-1 on tumor infiltrating lymphocytes (TIL) and PD-L1 on tumor cells.

**Results:**

Seven patients (38.9%) in cohort 1 showed progressive disease in both primary and intracranial lesions. Although the other 11 patients exhibited a partial response or stable disease in the primary lesion, eight showed a progression in BM. Interestingly, PD-1^+^ TILs were significantly decreased in BM (*P* = 0.034). For fifteen patients with adenocarcinoma, more distinctive patterns were observed in CD3^+^ (*P* = 0.078), CD8^+^ (*P* = 0.055), FOXP3^+^ (*P* = 0.016), and PD-1^+^ (*P* = 0.016) TILs.

**Conclusions:**

There may be discordant responses to an ICI of lung cancer between primary lung lesion and BM based on discrepancies in the tumor microenvironment. The diminished infiltration of PD-1^+^ TILs in tumor tissue within the brain may be one of the major factors that hinder the response to anti–PD-1 antibody in BM.

**Electronic supplementary material:**

The online version of this article (10.1186/s12885-018-5214-8) contains supplementary material, which is available to authorized users.

## Background

About 30% of advanced lung cancer patients develop brain metastases (BM), and their prognosis as well as quality of life are generally poor [1, 2]. The standard management for these patients includes metastasectomy and radiotherapy—either stereotactic radiosurgery or whole brain radiotherapy (WBRT). However, despite an improved local control rate [3], such localized treatments have inevitable toxic effects, including cognitive decline or a deterioration in the patient’s quality of life [4–6]. Furthermore, local treatment can delay the initiation of systemic treatment, which is crucial in patients with rapidly progressive disease [7, 8]. Accordingly, systemic treatment is recommended for patients with BM who are asymptomatic or experience only minimal neurological symptoms; this may provide a benefit for BM while simultaneously treating extracranial disease [9].

Recently, immune checkpoint inhibitors (ICIs) have emerged as a promising new treatment in various cancer types. These drugs, including anti–cytotoxic T-lymphocyte antigen 4 (CTLA-4) antibodies and anti–programmed cell death-1 (anti-PD-1)/programmed cell death ligand-1 (PD-L1) antibodies, reactivate the anti-tumor immunity of T cells [10, 11]. Although the efficacy as a first-line treatment in metastatic non-small-cell lung cancer (NSCLC) was not evident yet, [12] nivolumab have been approved by the US Food and Drug Administration (FDA) for the treatment of metastatic NSCLC and has become standard treatment in a second-line setting. [13–17]. However, most of these trials have excluded patients with any history of BM or active/symptomatic BM requiring steroid treatment. Furthermore, the evidence for immune checkpoint inhibitors in the management of BM has been largely limited to retrospective analyses of melanoma metastases and ipilimumab [18]. Therefore, the intracranial efficacy of ICIs is relatively unknown and has not yet been validated.

In this regard, prospective clinical trials of ICIs are currently under way, and tentative results suggested activity [18]. Recent early phase clinical trials have conducted to investigate the therapeutic efficacy of ICIs on BM in patients with NSCLC or melanoma [19, 20]. Although they reported that pembrolizumab and ipilimumab can have therapeutic activity on BM in patients with NSCLC and melanoma, many patients did not showed response in the brain or in extracranial sites. Further more, because these studies mainly included patients without neurological symptoms or the need for corticosteroids, the efficacy of ICIs on BM in real world are still inconclusive yet. Additional studies addressing combination treatment strategies and biomarker development are necessarily warranted.

Over the past decade, the tumor microenvironment (TME) has emerged as a critical regulator determining the response of ICIs [21]. Because of the distinctive composition of the extracellular matrix (ECM) as well as immunological environments in the brain, the TME of the brain has unique features that distinguishes it from the TME in primary lung cancer [22]. For example, unusual tissue-resident cell types, including microglia, astrocytes and neurons, are present in the brain’s TME [23], Furthermore, more importantly, the brain is one of the immune privileged organs that must be sheltered from immune cell entry and/or attack. Therefore, the TME of an early-stage brain tumor is generally immunosuppressive with essentially no trafficking or patrolling by peripheral immune cells [23]. Because immuno-oncology strategies rely on the sophisticated interaction between the tumor and its microenvironment, such a peculiar immunological environment of the brain presents a formidable challenge to overcome.

In the era of immunotherapy, understanding how BM are influenced by the immunological peculiarities of the brain will be crucial to forging therapeutic advances in lung cancer. The purposes of this study are 1) to compare intracranial and extracranial responses to ICIs in lung cancer patients with BM, and 2) to investigate differences in the TME between lung and brain metastases, focusing on the PD-1/PD-L1 pathway.

## Methods

### Patients and samples

Two independent cohorts of lung cancer patients were retrospectively analyzed to investigate the differences between primary tumors and BM in two respects: cohort 1 for comparing extra/intracranial responses to anti–PD-1 antibody; cohort 2 for comparing differences in the immunological TME. Cohort 1 included advanced lung cancer patients who were treated with intravenous nivolumab or pembrolizumab as part of an expanded access program (EAP), Keynote 010 trial (NCT01905657), or in routine practice from February 2014 to November 2016 at Seoul National University Hospital (SNUH) and Seoul National University Bundang Hospital. Treatment continued until disease progression, unacceptable toxicity that precluded continuing drug treatment, or death. Patients were allowed to continue treatment despite disease progression if they were deriving a clinical benefit according to an investigator’s assessment. Patients were eligible for treatment if they had at least one BM, with the longest diameter being ≥5 mm, before or during treatment with anti–PD-1 antibody. Patients who received local treatment for previously known BM were not excluded. Cohort 2 included lung cancer patients who underwent both brain metastasectomies for their BM, and primary lung surgery. Therefore, patients who were available for paired lung cancer and BM surgical specimens were enrolled. The Tissue Registry at SNUH was searched between June 2011 and November 2016. Two expert pathologists (S.H.K. and Y.K.J) reviewed tissue sections for adequacy. This study was approved by the SNUH Institutional Review Board (IRB approval number: H-1702-158-836) and was conducted in accordance with Declaration of Helsinki provisions.

### Response evaluation

A systemic response to anti–PD-1 antibody was measured by standard Response Evaluation Criteria in Solid Tumors (RECIST; version 1.1). An intracranial response was assessed by brain gadolinium-enhanced magnetic resonance imaging (MRI), using RECIST modified to allow target central nerve system lesions, 5 mm or larger in maximum diameter, and with up to five BMs permitted (modified RECIST) [24]. Representative MRI images for response evaluations are available in Additional file 1: Figure S1.

### Immunohistochemistry

Formalin-fixed paraffin-embedded specimens were examined by immuno-staining tumor cells and TILs. Immunohistochemistry (IHC) was performed using the following antibodies: rabbit anti–PD-L1 (E1L3N) XP® mAb (Cell Signaling Technology, Danvers, MA, USA), mouse anti-CD3 mAb (DAKO, Santa Clara, CA, USA), mouse anti-CD4 mAb (Cell Marque, Rocklin, CA, USA), rabbit anti-CD8 mAb (Thermo Scientific Fischer, Rockford, IL, USA), mouse anti–PD-1 mAb (Cell Marque, Rocklin, CA, USA) and mouse anti-FOXP3 mAb (Abcam, Cambridge, UK). IHC for TILs, except for CD8^+^ TILs, was performed using a Benchmark XT autostainer (Ventana Medical Systems, Basel, Switzerland). In the case of CD8^+^ IHC, a Bond-Max automated immunostainer (Leica Microsystems, Melbourne, Australia) was used. PD-L1 IHC was evaluated based on the intensity and proportion of membranous staining, with or without cytoplasmic staining, in tumor cells and was scored as follows: 0+ (no appreciable staining above background), 1+ (weak membranous staining and/or cytoplasmic staining), 2+ (moderate membranous staining and/or cytoplasmic staining), and 3+ (strong membranous staining and/or cytoplasmic staining) [25]. Each score was multiplied by the percentage of cells (0–100%) to achieve a H-score; H-score = (% of cells 3+)×3 + (% of cells 2+)×2 + (% of cells 1+). All slides were blinded with respect to clinical characteristics and outcomes, and were reviewed and scored by two experienced pathologists (S.H.K. and, Y.K.J.).

### Automated enumeration of tumor-infiltrating lymphocytes

To determine the amount of CD3^+^, CD4^+^, CD8^+^, PD-1^+^ and FOXP3^+^ TILs, representative slides were immunostained for CD3, CD4, CD8, PD-1 and FOXP3 and scanned for virtual microscopy using an Aperio ScanScope (Aperio Technologies, Vista, CA, USA). The number of TILs was automatically counted in intact tumor areas lacking necrosis using modified nuclear IHC algorithms in Aperio ImageScope software, as previously described [25]. The mean value of TILs per unit area (mm^2^) was calculated.

### Statistical analysis

Extra- and intracranial responses to anti–PD-1 antibody were descriptively analyzed. Differences in IHC markers between primary and BM were assessed using the paired Wilcoxon rank-sum test. Correlations between IHC markers were evaluated by Spearman’s rank correlation analysis. Survival analyses were performed for cohort 2 using the Kaplan–Meier method and were compared using a log-rank test. Overall survival (OS) was measured from the date of brain metastasectomy until either death due to any cause or the last follow-up date. A Cox proportional hazard regression model was applied to determine the hazard ratio (HR) for specific variables with respect to OS. For all statistical analyses, two-sided *P*-values *<* 0.05 were considered statistically significant. All statistical analyses were carried out using R version 3.3.2 (http://www.r-project.org).

## Results

### Demographics of study subjects

The clinicodemographic characteristics of patients in the two study cohorts are summarized in Table 1. Cohort 1 included 18 lung cancer patients who had BM before or during anti–PD-1 treatment. Most patients had an adenocarcinoma histology (66.7%), and stages IIIA–IV disease (83.3%). Three patients (16.7%) received cerebral irradiation by WBRT during treatment with an ICI. Cohort 2 included 20 lung cancer patients who underwent brain metastasectomy, and therefore paired primary and BM specimens were available. Seven pairs (35.0%) were obtained from cases with synchronous disease at their initial diagnosis, and 13 pairs (65.0%) were obtained from patients with metachronous disease who underwent metastasectomy 5 or more months after the initial diagnosis. The median interval between acquisitions of paired lesions was 17 months (range 0–81 months). No patient had been exposed to anti–PD-1 antibody in cohort 2, and five patients (25.0%) underwent cerebral irradiation before the acquisition of specimens.

**Table 1:** Clinicodemographic characteristics of patients in cohorts 1 and 2. Cohort 1 consisted of 18 lung cancer patients who underwent brain metastasectomy for metastatic brain tumors, while cohort 2 included 20 lung cancer patients with brain metastases who were treated with anti–PD-1 antibody (nivolumab or pembrolizumab). The two patient cohorts were independent of each other

Characteristics	Cohort 1 (*N* = 18)	Cohort 2 (*N* = 20)
Age, median (range)	59 (32–76)	60 (33–77)
Sex, n (%)		
Male	12 (66.7)	10 (50)
Female	6 (33.3)	10 (50)
Smoking, n (%)		
Current smoker	4 (22.2)	7 (35.0)
Ex-smoker	6 (33.3)	3 (15.0)
Never smoked	8 (44.4)	10 (50.0)
Stage at diagnosis^a)^, n (%)		
IA–IIB	1 (5.6)	4 (20.0)
IIIA–IV	15 (83.3)	16 (80.0)
Extensive disease (for small cell lung cancer)	2 (11.1)	0 (0.0)
Histology, n (%)		
Adenocarcinoma	12 (66.7)	15 (75.0)
Squamous cell carcinoma	1 (5.6)	4 (20.0)
Sarcomatoid carcinoma	2 (11.1)	1 (5.0)
Small cell lung cancer	2 (11.1)	0 (0.0)
Mutational status, n (%)		
EGFR	3 (16.7)	9 (45.0)
ALK	2 (11.1)	0 (0.0)
Temporal relationship, n (%)		
Synchronous	NA	7 (35.0)
Metachronous	NA	13 (65.0)
Anti–PD-1 antibody treatment		
Nivolumab	14 (77.8)	NA
Pembrolizumab	4 (22.2)	NA
No. of chemotherapy treatments before EAP or brain surgery, n (%)		
0	0 (0.0)	8 (40.0)
1	6 (33.3)	7 (35.0)
2	7 (38.9)	2 (10.0)
≥ 3	5 (27.8)	3 (15.0)
Cerebral irradiation before EAP or brain surgery, n (%)		
Whole brain radiotherapy	3 (16.7)	2 (10.0)
Radiosurgery	4 (22.2)	2 (10.0)
Both	3 (16.7)	1 (5.0)
Cerebral irradiation during EAP, n (%)		
Whole brain radiotherapy	3 (16.7)	NA
Radiosurgery	0 (0.0)	NA
Both	0 (0.0)	NA

^a^TNM classification according to the 7th edition of the AJCC cancer staging manual

Abbreviations: *EGFR* Epidermal growth factor receptor, *ALK* anaplastic lymphoma kinase, *PD-1* programmed cell death-1, *AJCC* American Joint Committee on Cancer, *EAP* expanded access program

### Analysis of cohort 1: Comparison of response to anti–PD-1 antibody between primary lung cancer and brain metastasis

**Fig. 1:**
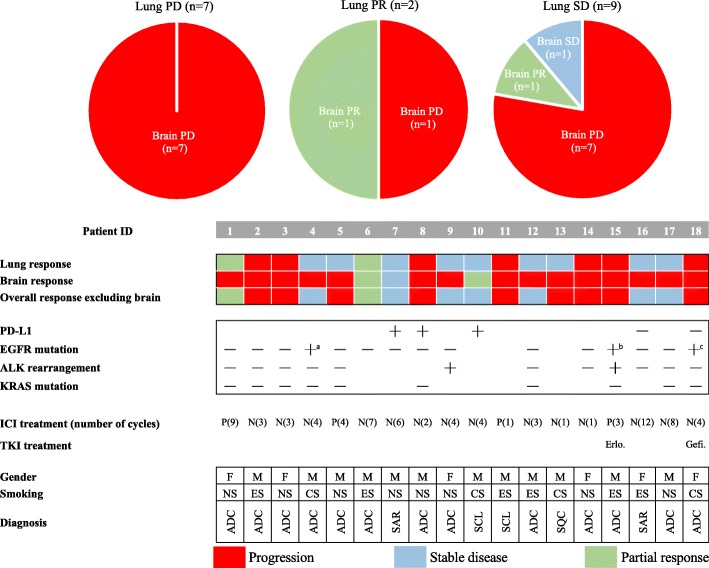
Treatment response to anti–PD-1 antibody in cohort 1. (Upper) Pie charts demonstrating the response in primary lung cancer and paired brain metastases. (Lower) The treatment response along with clinicodemographic characteristics of individual patients in cohort 1. Each patient received either nivolumab (N) or pembrolizumab (P) for the specified number of cycles. Each tile in this figure denotes the treatment response: red for progressive disease, blue for stable disease, and green for a partial response. Abbreviations: PD, progressive disease; PR, partial response; SD, stable disease; ID, identification number; P, pembrolizumab; N, nivolumab; PD-L1, programmed cell death ligand-1; PD-1, programmed cell death-1; M, male; F, female; EGFR, epidermal growth factor receptor; ALK, anaplastic lymphoma kinase; NS, never smoked; ES, ex-smoker; CS, current smoker; ADC, adenocarcinoma; SAR, pulmonary sarcomatoid carcinoma; SCL, small-cell lung cancer; SQC, squamous cell carcinoma, Erlo., erlotinib; Gefi., gefitinib; ICI, immune checkpoint inhibitor; TKI, tyrosine kinase inhibitor. a: Exon 20 insertion. b: Exon 19 deletion. c: L858R mutation

Of 18 patients in cohort 1, the primary lung lesion had progressed in seven patients (38.9%). All such patients showed progressive disease in intracranial lesions (Fig. 1). The primary lesion showed a stable disease (*n* = 9, 50.0%) and partial response (*n* = 2, 11.1%) in 11 patients, with eight of these exhibiting progressive disease in BM. Although two patients showed a partial response in brain tumors, they were found to have received WBRT with nivolumab. Collectively, these results implied that BM was poorly responsive to anti–PD-1 antibody compared with the primary lung lesion.

### Analysis of cohort 2: Comparison of immunological tumor microenvironment between primary lung cancer and brain metastasis

**Fig. 2:**
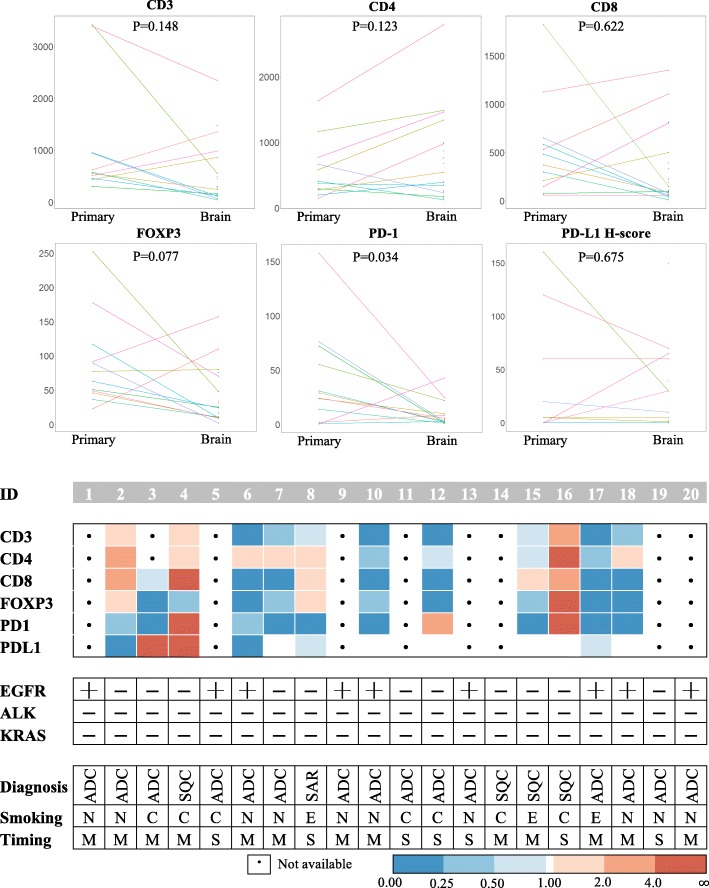
Immunohistochemical analysis of CD3, CD4, CD8, FOXP3, and PD-1 on tumor-infiltrating lymphocytes, and PD-L1 on tumor cells of patients in cohort 2. (Upper) Ladder plots demonstrate the different expression of each marker between primary lung cancer and brain metastases. Individual patients in cohort 2 are denoted as different colored lines. Statistical significance was estimated using a paired Wilcoxon rank-sum test. (Lower) Detailed information, including clinicodemographic characteristics, are summarized in this figure. Because lung biopsy tissues from 8 patients (patients 1, 5, 9, 11, 13, 14, 19, 20) were too small to be examined by IHC, paired analysis was possible in only 12 out of 20 patients in cohort 2. Each colored tile in this figure represents the increased expression of each marker in the brain relative to that in primary lung cancers. Abbreviations: PD-1, programmed cell death-1; PD-L1, programmed cell death ligand-1; ID, identification number; EGFR, epidermal growth factor receptor; ALK, anaplastic lymphoma kinase; Erlo., erlotinib; Gefi., gefitinib; TKI, tyrosine kinase inhibitor; ADC, adenocarcinoma; SQC, squamous cell carcinoma; SAR, pulmonary sarcomatoid carcinoma; N, never smoked; C, current smoker; E, ex-smoker; M, metachronous; S, synchronous. a: Exon 19 deletion. b: L858R mutation

**Fig. 3:**
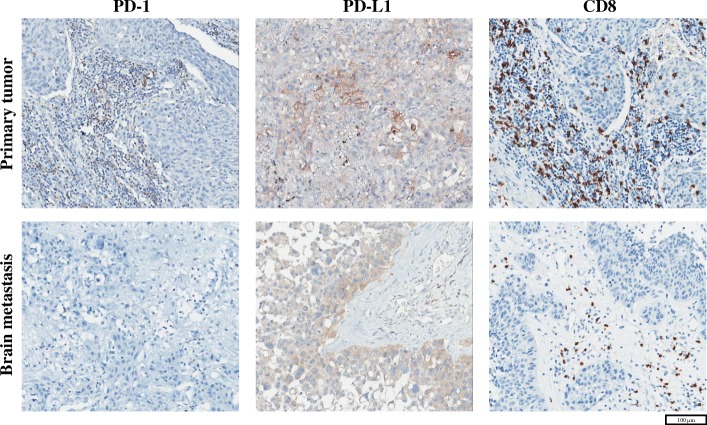
Representative immunohistochemical images of PD-1, PD-L1, and CD8 in primary lung cancer and brain metastatic tissues of a patient from cohort 2 (patient ID 17). Abbreviations: PD-1, programmed cell death-1; PD-L1, programmed cell death ligand-1

On the hypothesis that the distinctive immunological TME of the brain determines the response to the anti–PD-1 antibody, the amounts of TILs and PD-L1 expression on tumor cells in the brain TME were compared with those of primary lung cancer specimens (Fig. 2). Only twelve (60.0%) out of 20 patients in cohort 2 were eligible for a paired comparison because lung biopsy specimens obtained from the other eight patients were too small to evaluate the TME; Three of them had synchronous BM, while the others had metachronous BM. The median interval between acquisitions of paired lesions in patients metachronous BM was 23.4 months (range 4.7–81.5 months). The eight patients were not excluded from cohort 2 because they were eligible for survival analysis. The amounts of CD3^+^, CD4^+^, and CD8^+^ TILs, and the PD-L1 H-score of tumor cells did not show statistically significant differences between primary lung cancers and BM tissues, with *P* values of 0.148, 0.123, 0.622 and 0.675, respectively. On the other hand, FOXP3^+^ TILs showed a decreasing tendency in the intracranial lesion (*P* = 0.077), and, more interestingly, PD-1^+^ TILs decreased in the brain in a statistically significant manner (*P* = 0.034). For fifteen patients with adenocarcinoma (75.0%), more distinctive patterns were observed in CD3^+^ (*P* = 0.078), CD8^+^ (*P* = 0.055), FOXP^+^ (*P* = 0.016), and PD1^+^ (*P* = 0.016) TILs (Additional file 2: Figure S2). Figure 3 shows representative images of a male patient (ID 17 of cohort 2) with adenocarcinoma histology. In the BM specimen of the patient, PD-1^+^ and CD8^+^ TILs were dramatically decreased, while PD-L1 expression on tumor cells was relatively unchanged. The infiltration of CD3^+^ TILs correlated positively with the PD-L1 H-score of tumor cells in the primary lung cancer (Spearman ρ = 0.545, *P* = 0.083; Additional file 3: Figure S3A), and BM (Spearman ρ = 0.444, *P* = 0.049; Additional file 3: Figure S3B). No correlation between PD1+ TILs and PD-L1 expression was noted in the primary lung cancer Spearman ρ = 0.116, *P* = 0.720; Additional file 3: Figure S3C). In contrast, the infiltration of PD1+ TILs positively correlated with PD-L1 expression of tumor cells in BM (Spearman ρ = 0.550, *P* = 0.012; Additional file 3: Figure S3D). For patients with metachronous disease, there was no significant correlation between PD-1^+^ TIL infiltration on brain TME and the interval of development of BM. (Spearman ρ = 0.133, *P* = 0.744; Additional file 4: Figure S4). Altogether, these results revealed that the brain TME differed from that of the primary cancer in terms of an immunological environment, with a markedly decreased amount of PD-1^+^ TILs. The infiltration of CD3^+^ and CD8^+^ TILs was also decreased, especially for lung adenocarcinoma.

### Prognostic implication of immunological tumor microenvironment in metastatic brain tumors

**Fig. 4:**
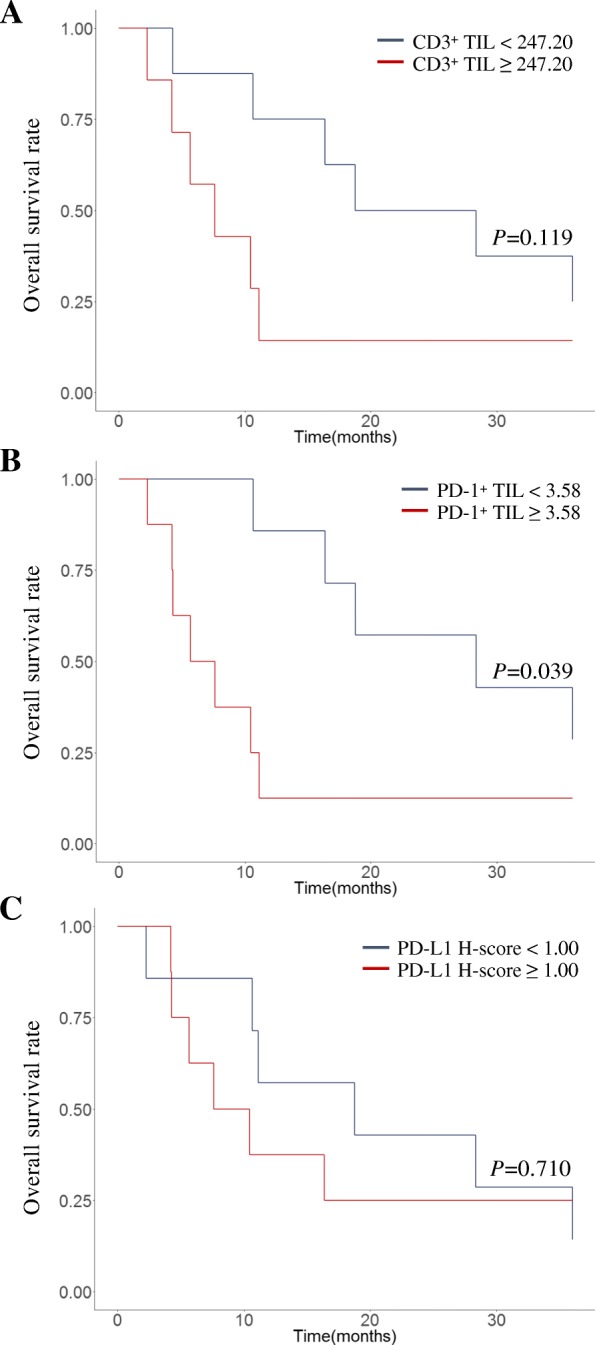
Kaplan–Meier plots for lung cancer patients with adenocarcinoma histology in cohort 2 according to **a** the amount of CD3^+^ TILs, **b** PD1^+^ TILs, and **c** the PD-L1 H-score in brain metastatic tissue. Overall survival was measured from the date of surgery until either death due to any cause or the last follow-up date. The cutoff-points were arbitrarily set to the median value of each variable. The significance of differences in survival curves were compared using a log-rank test. Abbreviations: TIL, tumor-infiltrating lymphocyte; PD-1, programmed cell death-1; PD-L1, programmed cell death ligand-1

To investigate the prognostic significance of immunological TME of brain metastasis, the OS rate of cohort 2 after brain surgery was studied in terms of the amount of CD3^+^ and PD1^+^ TILs, and PD-L1 expression on tumor cells. Since there were no clear-cut off points for these values, the median of each variable was arbitrarily used as a cut-off point. All patients in cohort 2 did not exhibit any survival differences (data not shown). For adenocarcinoma patients in cohort 2, however, a higher infiltration of CD3^+^ TILs tended was associated with a worse prognosis (HR 2.47, 95% CI 0.77–7.96; *P* = 0.130, log-rank *P* = 0.119; Fig. 4a) Interestingly, patients showing a higher infiltration of PD1^+^ TILs exhibited a dismal prognosis that was statistically significant (HR, 1.20, 95% CI, 1.01–11.04; *P* = 0.049; log-rank *P* = 0.039; Fig. 4b). In terms of PD-L1 expression on tumor cells, there was no prognostic implication according to the PD-L1 H-score (HR 1.24, 95% CI 0.39–3.91; *P* = 0.711, log-rank *P* = 0.710; Fig. 4c). Collectively, this analysis showed that the immunological TME in the brain had a prognostic implication after brain metastasectomy, especially for patients with adenocarcinoma histology.

## Discussion

In this study, we found that the BM of lung cancer did not respond well to ICIs. Immunohistochemical analysis revealed a decreased infiltration of PD-1^+^ TILs in BM compared to primary lung cancer. These findings demonstrate that decreased PD-1^+^ of TILs could be one of the potential causative reasons for a poor intracranial response to ICIs.

Activated T cells have the potential to penetrate the blood–brain barrier (BBB) and readily infiltrate tumors within the brain, giving rise to the possibility that an ICI may have a therapeutic effect on BM [26]. However, the brain has been generally considered an immune privileged site that may dampen the therapeutic efficacy of ICIs because of the presence of BBB [27]. Although there has been no published pharmacokinetic or pharmacodynamics studies in on-treatment brain tissue to allow determination of drug penetration into the tumor, some preclinical data demonstrated ICI’s penetration to BBB for primary brain tumors and for metastatic tumors. [28] Furthermore, for melanoma patients with stable asymptomatic brain metastases, ipilimumab, and to a lesser extent pembrolizumab, did show any activity, with half of all patients achieving a partial response or stable disease [29]. Whether this also applies to lung cancer patients is currently being investigated in a number of early phase immunotherapy trials.

An recent interim analysis of a phase II study showed that pembrolizumab may have a role in NSCLC patients with brain metastases [19]. Unfortunately, however, many patients did not respond to pembrolizumab in the brain or in extracranial sites. Similarly, only 16.7% of the patients in cohort 1 of the present study responded to treatment for BM with anti–PD-1 antibody. Although two patients achieved a partial response, they underwent WBRT with anti–PD-1 antibody, making it difficult to interpret the ICI response. The superior response rate observed in the phase II study might be due to exclusion of patients with any history of BM or active/symptomatic BM. In general, there is huge gap between clinical trials and real world lung cancer patients because of strict criteria for patient enrollment of clinical trials. [30] Our study represents that the BM of lung cancer patient in real world do not seem to respond well to ICIs.

Over the past decades, the TME has been considered a fundamental regulator of cancer progression and therapeutic efficacy. The TME contains various noncancerous cell types, including endothelial cells, pericytes, fibroblasts, and immune cells [21]. While several of these cell types are common to both, there are significant differences between the TMEs of primary lung cancer and the brain, allowing mechanisms that regulate tumor cell niches in the brain to differ from those of other tissues [22, 23]. In order to explain why BM were not responsive to ICIs, attention was paid to the immunological environment in the TME of the brain. The brain TME was found to have lost PD-1^+^ TILs despite their presence in primary lung cancer specimens, which is a novel finding of this study. In accordance with others [31–33], adenocarcinoma patients displayed a more distinctive pattern of a low infiltration of both CD3^+^ and CD8^+^ TILs, suggesting that a significant number of patients lack any significant local immune response in association with their BM. Although PD-L1 expression of tumor cells showed no significant differences in this study, others have reported that BM lost PD-L1 expression that was normally present in primary lung cancer specimens [22, 34]. Overall, such an immunological heterogeneity may explain, at least in part, the different therapeutic responses to PD-1 inhibitors.

While limited data suggest that the intracranial response to ICIs may have the distinct advantage of producing durable responses in selected patients [19], there is no definitive biomarker to identify this population. Although there have been significant efforts to develop PD-L1 as a predictive biomarker to select patients for treatment with PD-1 or PD-L1 inhibitors, consensus criteria for evaluating PD-L1 as a predictive biomarker have not yet been settled [35]. As indicated in our study, the immunological TME in the brain, including the infiltration of PD-1^+^ TILs, may be important in predicting the clinical benefits of PD-1/PD-L1 checkpoint blockades in lung cancer patients with BM. Therefore, we suggest that the role of PD-1 as a biomarker in the brain TME should be investigated in future clinical studies of immunotherapy in lung cancer with BM. When physicians decide to treat patients with a PD-1 or PD-L1 inhibitor, the distinctive immunological environment of the brain should be taken into consideration.

This study has several limitations. The two cohorts analyzed in this study were small due to the limited number of patients with BM who underwent anti–PD-1 antibody treatment or a brain metastasectomy. Furthermore, since the two cohorts were independent of each other, the predictive role of PD-1 for anti–PD-1 antibody treatment could not be evaluated directly. However, it was sufficient to generate the hypothesis that PD-1 could be a potential biomarker. Actually, some of the progressive diseases observed in cohort 1 could have been pseudoprogressions. However, the main purpose of this study was to investigate the difference in treatment response between BM and primary lung cancer, rather than the absolute response of each organ. Therefore, the existence of pseudoprogression is unlikely to change the conclusion of this study.

## Conclusions

Our findings suggest that there may be discordant extra/intracranial responses to ICIs. The diminished infiltration of PD-1^+^ TILs into tumor tissue in the brain may be one of potential factors that hinders the response to anti-PD-1 antibody in the BM. These findings provide a rationale for studying the infiltration of PD-1^+^ TILs in the brain as a potential biomarker for future immunotherapy trials that incorporate patients with BM. Hopefully, ongoing studies will improve treatment selection strategies for lung cancer patients with BM.

## Additional files

Additional file 1: **Figure S1.** Representative images for response evaluation in brain metastasis. An intracranial response was assessed by brain gadolinium-enhanced magnetic resonance imaging, using Response Evaluation Criteria in Solid Tumors modified to allow target central nerve system lesions, 5 mm or larger in maximum diameter, and with up to five BMs permitted (modified RECIST). Each patient received either nivolumab (N) or pembrolizumab (P) for the specified number of cycles. Abbreviations: ADC, adenocarcinoma; SCL, small-cell lung cancer; SAR, pulmonary sarcomatoid carcinoma; P, pembrolizumab; N, nivolumab; PD, progressive disease; PR, partial response; SD, stable disease. (TIF 2970 kb)

Additional file 2: **Figure S2.** Immunohistochemical analysis of CD3, CD4, CD8, FOXP3, and PD-1 on tumor-infiltrating lymphocytes, and PD-L1 on tumor cells of patients with an adenocarcinoma histology in cohort 2. Ladder plots demonstrate the different expression of each marker between primary lung cancer and brain metastases. Individual patients are denoted as different colored lines. Statistical significance was estimated using a paired Wilcoxon rank sum test. (TIF 497 kb)

Additional file 3: **Figure S3.** Scatter plots demonstrating the correlation between PD-L1 expression on tumor cells and the amount of CD3^*+*^ TILs or PD1^+^ TILs in primary lung cancer specimens, and metastatic brain tumors. Correlation was evaluated by Spearman’s rank correlation analysis. Abbreviations: PD-L1, programmed cell death ligand-1; TIL, tumor-infiltrating lymphocyte. (TIF 379 kb)

Additional file 4: **Figure S4.** Scatter plot demonstrating the correlation between infiltrating PD-1^+^ tumor-infiltrating lymphocyte on brain metastasis and the interval of development of brain metastasis. Correlation was evaluated by Spearman’s rank correlation analysis. Abbreviations: PD-1, programmed cell death-1; TIL, tumor-infiltrating lymphocyte. (TIF 143 kb)

## Abbreviations

ADC: Adenocarcinoma
ALK: Anaplastic lymphoma kinase
EGFR: Epidermal growth factor receptor
ID: Identification number
N: Nivolumab
P: Pembrolizumab
PD: Progressive disease
PD-1: Programmed cell death-1
PD-L1: Programmed cell death ligand-1
PR: Partial response
SAR: Pulmonary sarcomatoid carcinoma
SCL: Small-cell lung cancer
SD: Stable disease
SQC: Squamous cell carcinoma
TIL: Tumor-infiltrating lymphocyte
