# Supplementary figures and images for: Differences in tumor microenvironments between primary lung tumors and brain metastases in lung cancer patients: therapeutic implications for immune checkpoint inhibitors
Source: BMC Cancer. 2019 Jan 7;19:19. doi: 10.1186/s12885-018-5214-8 (PMC6322302; doi:10.1186/s12885-018-5214-8)

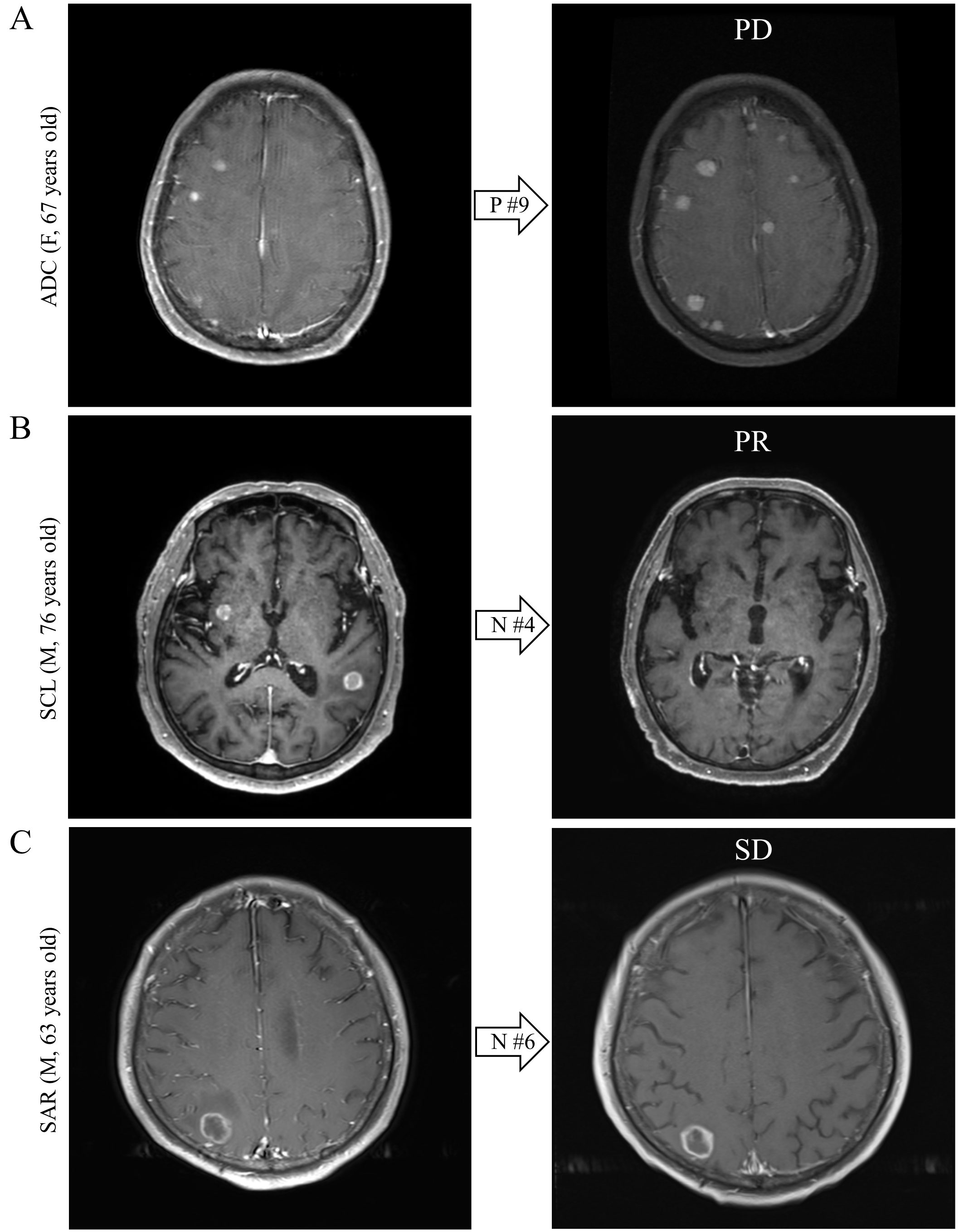

Supplement: Supplementary file 1 — Figure S1. Representative images for response evaluation in brain metastasis. An intracranial response was assessed by brain gadolinium-enhanced magnetic resonance imaging, using Response Evaluation Criteria in Solid Tumors modified to allow target central nerve system lesions, 5 mm or larger in maximum diameter, and with up to five BMs permitted (modified RECIST). Each patient received either nivolumab (N) or pembrolizumab (P) for the specified number of cycles. Abbreviations: ADC, adenocarcinoma; SCL, small-cell lung cancer; SAR, pulmonary sarcomatoid carcinoma; P, pembrolizumab; N, nivolumab; PD, progressive disease; PR, partial response; SD, stable disease. (TIF 2970 kb) [file 12885_2018_5214_MOESM1_ESM.tif]

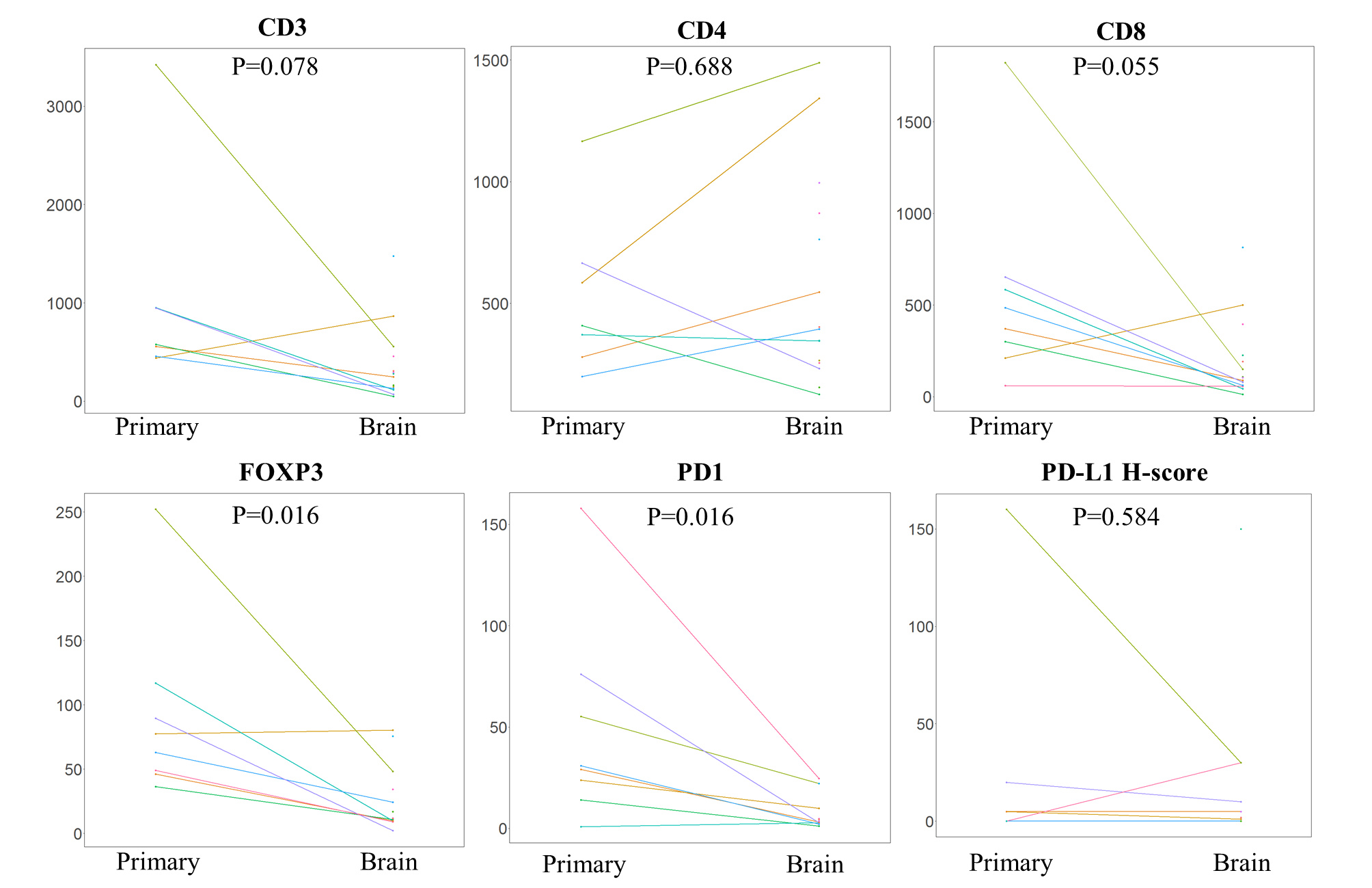

Supplement: Supplementary file 2 — Figure S2. Immunohistochemical analysis of CD3, CD4, CD8, FOXP3, and PD-1 on tumor-infiltrating lymphocytes, and PD-L1 on tumor cells of patients with an adenocarcinoma histology in cohort 2. Ladder plots demonstrate the different expression of each marker between primary lung cancer and brain metastases. Individual patients are denoted as different colored lines. Statistical significance was estimated using a paired Wilcoxon rank sum test. (TIF 497 kb) [file 12885_2018_5214_MOESM2_ESM.tif]

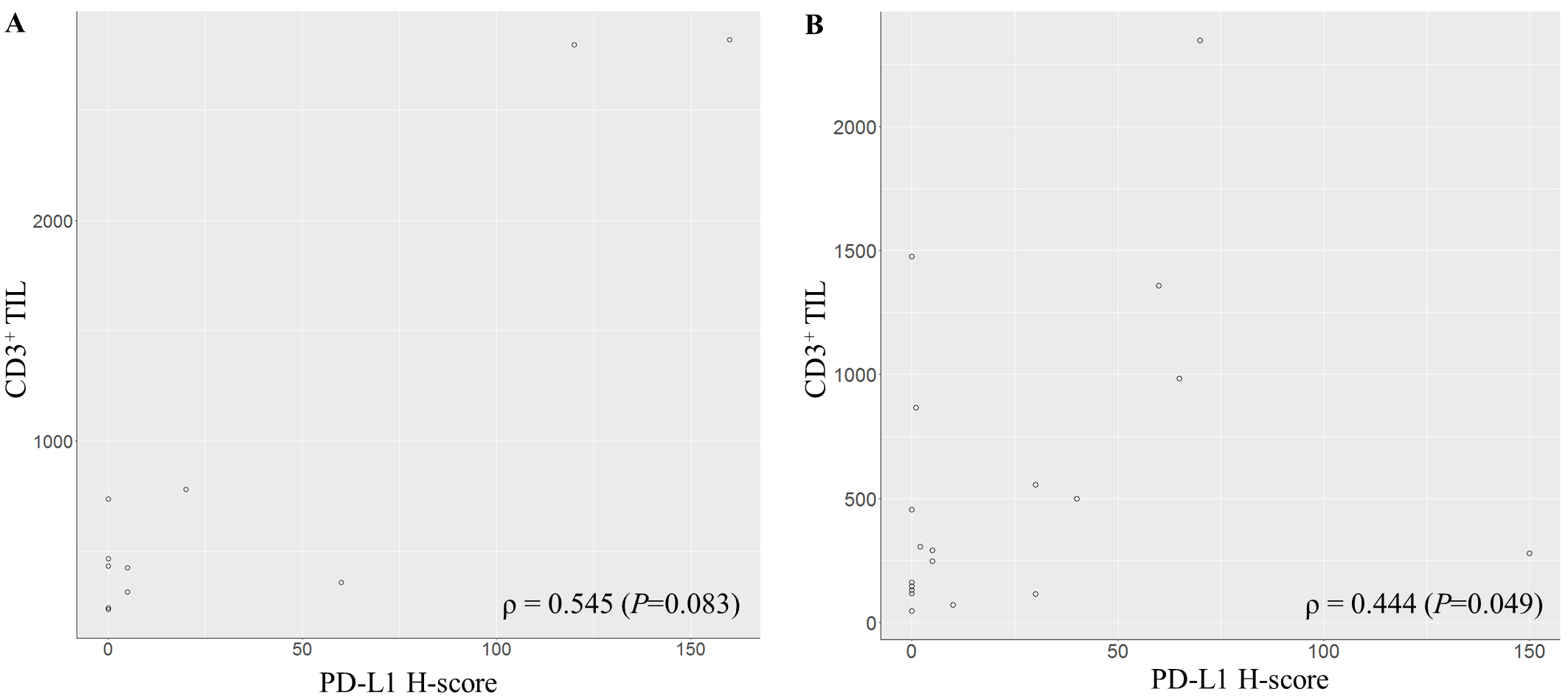

Supplement: Supplementary file 3 — Figure S3. Scatter plots demonstrating the correlation between PD-L1 expression on tumor cells and the amount of CD3+ TILs or PD1+ TILs in primary lung cancer specimens, and metastatic brain tumors. Correlation was evaluated by Spearman’s rank correlation analysis. Abbreviations: PD-L1, programmed cell death ligand-1; TIL, tumor-infiltrating lymphocyte. (TIF 379 kb) [file 12885_2018_5214_MOESM3_ESM.tif]

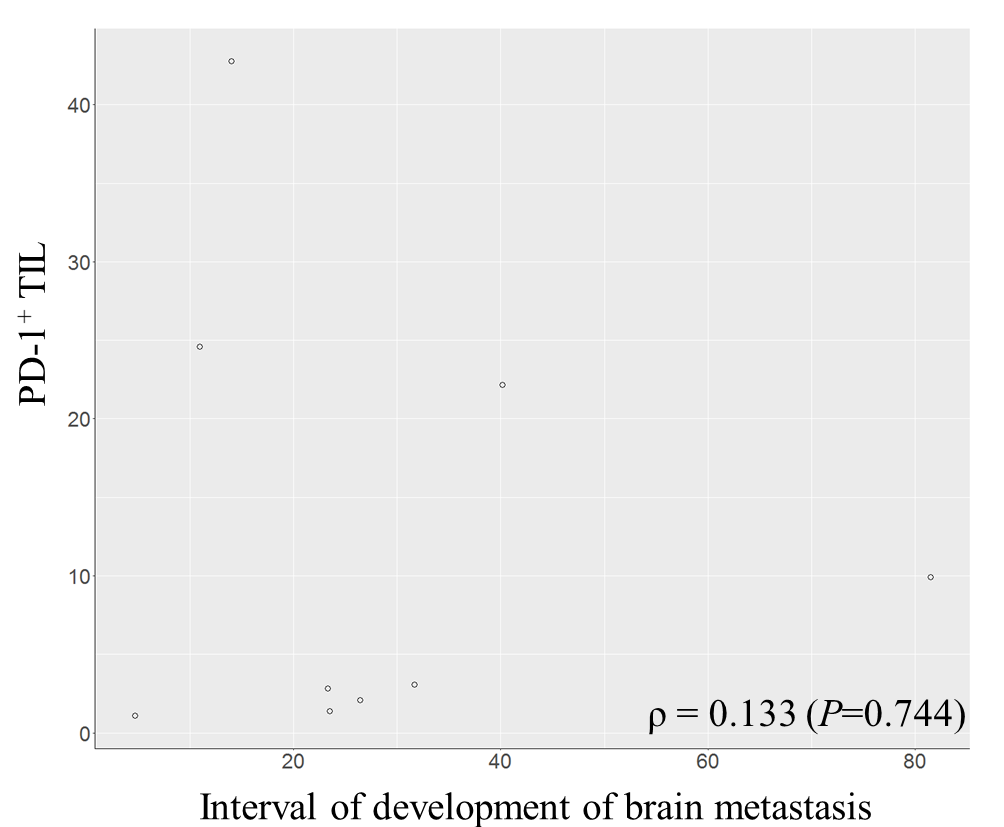

Supplement: Supplementary file 4 — Figure S4. Scatter plot demonstrating the correlation between infiltrating PD-1+ tumor-infiltrating lymphocyte on brain metastasis and the interval of development of brain metastasis. Correlation was evaluated by Spearman’s rank correlation analysis. Abbreviations: PD-1, programmed cell death-1; TIL, tumor-infiltrating lymphocyte. (TIF 143 kb) [file 12885_2018_5214_MOESM4_ESM.tif]
